# Neurodiversity in Saudi Arabia: Towards quality education and reduced inequalities

**DOI:** 10.12688/f1000research.155002.2

**Published:** 2024-12-09

**Authors:** Ahmed Yahya Almakrob, Ahmed Alduais, Alex S. M. Mhone, Borey Be

**Affiliations:** 1 Department of English, College of Sciences and Humanities, Prince Sattam bin Abdulaziz University, Al Kharj, Riyadh Province, Saudi Arabia; 2 Ibb University, Ibb, Ibb Governorate, Yemen; 3 Department of Education, The Catholic University of Malawi, Limbe, Southern Region, Malawi; 4 College of Education, The University of Cambodia, Phnom Penh, Phnom Penh, Cambodia

**Keywords:** Neurodiversity, Special Education Laws, Saudi Arabia, Inclusive Education, Sustainable Development Goals, Quality Education, Reduced Inequalities

## Abstract

Traditional educational frameworks in Saudi Arabia have historically adopted a deficit-based approach to special education, potentially overlooking the benefits of neurodiversity. As global educational paradigms shift toward inclusive practices, examining the alignment of Saudi special education laws with neurodiversity principles becomes crucial. This content analysis study aimed to explore the existing literature on special education in Saudi Arabia to ascertain whether the laws and policies support or hinder the practice of neurodiversity, a concept that is gaining international recognition but remains nascent in Saudi Arabia. A systematic literature search was performed in Web of Science and Scopus, yielding 21 relevant studies after screening and application of inclusion criteria. A content analysis was conducted, focusing on the categorization of data relevant to special education laws and their implications for neurodiversity. The analysis identified seven key categories, including Implementation of Inclusive education and transition services, that reflect the current state of special education laws in relation to neurodiversity. A conceptual model was developed, illustrating the potential of special education laws to both support and impede neurodiversity in Saudi Arabia and potentially worldwide.

## Introduction

### Neurodiversity

Neurodiversity acknowledges the intrinsic variation in human cerebral architecture, encompassing a spectrum of sociability, cognition, attention, and emotional states, suggesting an inherent appreciation for such neurological differences ( Armstrong, 2010). Armstrong posits the fallacy of a ‘standard’ brain, arguing for the recognition of cerebral diversity as a parallel to the richness found in ecological and cultural variance, ultimately framing it as advantageous rather than detrimental ( Armstrong, 2010). Conversely, Weiss (2022) conceptualizes neurodiversity as an acceptance of brain function and behavioral differences, considering them a natural facet of human variation. This perspective necessitates embracing cognitive diversity to foster societal cohesiveness. The term ‘neurodivergence’ encapsulates a collective of individuals whose cognitive function diverges from the Predominant Neurotype (PNT), including those with Autism, ADHD, Dyslexia, Dyspraxia/DCD, Dyscalculia, Tourette’s Syndrome, Developmental Language Disorder, and various speech and auditory processing conditions ( Sandland et al., 2023).

The interplay between the concept of neurodiversity and special education legislation is pivotal in shaping the educational landscape for neurodivergent individuals. Special education laws, such as the Individuals with Disabilities Education Act (IDEA) in the United States, are designed to ensure that students with diverse cognitive profiles receive tailored educational opportunities. However, the efficacy of these laws in supporting or impeding neurodiversity hinges on their implementation. While they have the potential to affirm the principles of neurodiversity by promoting inclusive education and individualized support, there is an ongoing debate regarding the extent to which these laws inadvertently contribute to the stigmatization and segregation of neurodivergent individuals, potentially clashing with the ethos of neurodiversity that Armstrong (2010) and Weiss (2022) advocate. Thus, the interpretation and application of special education laws play a crucial role in either reinforcing or challenging societal perceptions of neurodiversity.

### Special Education in Saudi Arabia

Since its inception in 1958, special education in Saudi Arabia has witnessed substantial advancements in the provision of services for students with disabilities. However, improvement is still required in certain areas. Initially, the domain of special education was solely concerned with providing instruction to students who were able to learn despite intellectual disabilities, severe visual impairments, or auditory impairments through special schools. These establishments are presently not regarded as the most suitable environments for exceptionally talented students. The integration of students with disabilities into general public institutions, nevertheless, served as a manifestation of this emerging paradigm and conviction ( Bin Battal, 2016).

The special education has made considerable progress in delivering services to students with disabilities ( Aldabas, 2015). Nonetheless, the need for educational and social inclusion of children with intellectual disabilities continues to grow throughout the world. Factors contributing to the increasing need include family advocacy activities, shifting attitudes about the nature of impairment, and increased international acknowledgment of the rights of children with disabilities. In the 1970s, Saudi Arabia began investing in educational programs and support for students with disabilities in both segregated and mainstream education settings ( Abu-Alghayth et al., 2022).

The term “Special Institutes” or “Special Education Institutes”, as they are known in Saudi Arabia, refers to separate schools, special schools, or special education schools. These schools include schools for the blind, schools for the deaf, and schools for the mentally retarded ( UNESCO, 2010). Special education services for students with disabilities have been introduced in response to the inadequacy of the current public education system, which is unable to fully accommodate many impaired students. Every country in the world has built its own particular education services programme and improves the education organisation on an annual basis. Saudi Arabia is one of the developing countries that has established and updated its educational model over time to allow children with disabilities to get an education ( Awdah Alzahrani, 2023).

### Current State of Special Education in Saudi Arabia

According to the Ministry of Education, the country’s education policy includes many essentials and factors related to special education. These include respecting the dignity of individuals and providing opportunities for them to develop their skills and abilities, so they can contribute to national development (Article 36). The policy also emphasizes taking care of students who are slow in their studies and working to eliminate the causes of their underdevelopment. It calls for the creation of temporary and permanent programs tailored to their needs (Article 55). Additionally, the policy highlights the importance of special education and care for mentally and physically impaired students. It aims to make education accessible to all children in accordance with Islamic teachings (Article 56). Lastly, the policy emphasizes the need to focus on education for physically and mentally disabled individuals, creating a special cultural curriculum and diversified training programs to meet their specific needs (Article 188) ( MOE, 2021).

Special education services in Saudi Arabia have received much attention over the past 15 years, with increased focus on offering such services ( Husain R Alruwaili, 2016). The country has a National Strategy for the Development of General Education, which affirms the provision of equal opportunities for people with disabilities ( MOE, 2021). Disability laws in Saudi Arabia mandate that higher education institutions provide support for special needs learners to guarantee educational opportunities equivalent to opportunities for their nondisabled peers ( Abed & Shackelford, 2020).

### Sustainable Development Goals (SDGs)

SDG 4 aims to guarantee inclusive and equitable quality education and promote lifelong learning opportunities for all. This includes attempts such as ensuring that all girls and boys complete free, equitable, and quality primary and secondary education, as well as giving access to excellent early childhood development and care. Additionally, it aspires to remove gender inequities in education and provide equitable access to all levels of education for vulnerable groups, including persons with disabilities and indigenous peoples ( United Nations, 2023a). Despite progress, as many as 48.1 per cent of girls remain out of school in some regions. Gender gaps in primary and secondary enrolment rates have nearly closed, on average. Yet 15 million girls are not in primary school right now, compared to 10 million boys. In adolescence, higher numbers of girls often drop out of secondary school for reasons including early pregnancy and the expectation that they should contribute to household work ( UN Women, n.d.).

On the other hand, SDG 10 focuses on reducing disparities within and across nations. This comprises enabling and encouraging the social, economic, and political inclusion of all individuals, irrespective of age, sex, impairment, race, ethnicity, origin, religion, or economic condition. It also tries to promote equal opportunity and eliminate disparities by removing discriminatory laws, policies, and practices ( United Nations, 2023b).

In Saudi Arabia, achieving SDG 4 (quality education) and SDG 10 (reduced inequalities) presents several challenges. SDG 4 addresses some concerns, including inequities in educational accessibility and quality, the effects of the COVID-19 epidemic on education, and the need for total educational reform. Saudi Arabia has faced challenges such as the impact of the COVID-19 pandemic on education, disparities in educational accessibility and quality, and the need for major education reform. Furthermore, the persistence of disparities in literacy rates, enrolment rates, and educational attainment, particularly in some areas, poses a challenge to implementing universal education goals ( Barry, 2019; Matilde, 2024; OECD, 2020; Reema et al., 2022). Regarding SDG 10, Saudi Arabia faces obstacles in tackling many types of inequality, including as income, gender, and disability. This necessitates equitable resource allocation, investments in education and skill development, and combatting discrimination. Saudi Arabia’s Gini Coefficient Index, which measures income distribution, was 45.6 in 2019, suggesting high income inequality ( Statista, 2024; World Economics, 2024). Furthermore, the country confronts challenges in resolving ongoing national inequalities, notably in terms of income and economic status ( Statista, 2024).

### Purpose of the present study

This content analysis study aims to explore and evaluate the existing published literature on special education within the Kingdom of Saudi Arabia, with a specific focus on examining how special education laws either enhance or impede the practice of neurodiversity—a concept that, while globally recognized, remains relatively nascent in the Saudi context. In advancing the discourse on neurodiversity, this study seeks to contribute to the burgeoning dialogue on inclusive education and the acknowledgment of diverse neurological conditions as a facet of human diversity rather than deficits. The scope of this study is confined to the examination of peer-reviewed articles, institutional reports, and legislative documents, all of which contribute to an understanding of the state of special education laws. Through content analysis, the study constructs a conceptual model that elucidates whether the current special education framework in Saudi Arabia is conducive to fostering an environment that embraces neurodiversity or if it inadvertently contributes to the marginalization of this population.

## Method

### Sample

The initial data were collected using a search strategy implemented on two major academic databases, Web of Science and Scopus. The search terms were selected to capture the breadth of literature on special education within the context of Saudi Arabia. The terms ‘special education policy’, ‘special education legislation’, ‘special education law’, and ‘special education regulation’ were used in conjunction with Boolean operators, searching within both titles and abstracts for an inclusive retrieval process. Additionally, the term ‘Saudi Arabia’ was included in the abstract search to narrow the focus geographically. This strategy yielded an initial pool of 112 studies. Since our focus was on the existing policies, laws, and legislations regarding neurodiversity, we used general search terms for the main concepts without searching for specific disorders or disabilities. This decision was also enhanced by our assumption that laws and legislations are always made for types of disabilities based on their categories (e.g., neurodevelopmental disorders, physical disability) rather than one by one (e.g., autism, hearing impairment).

**Figure 1.**
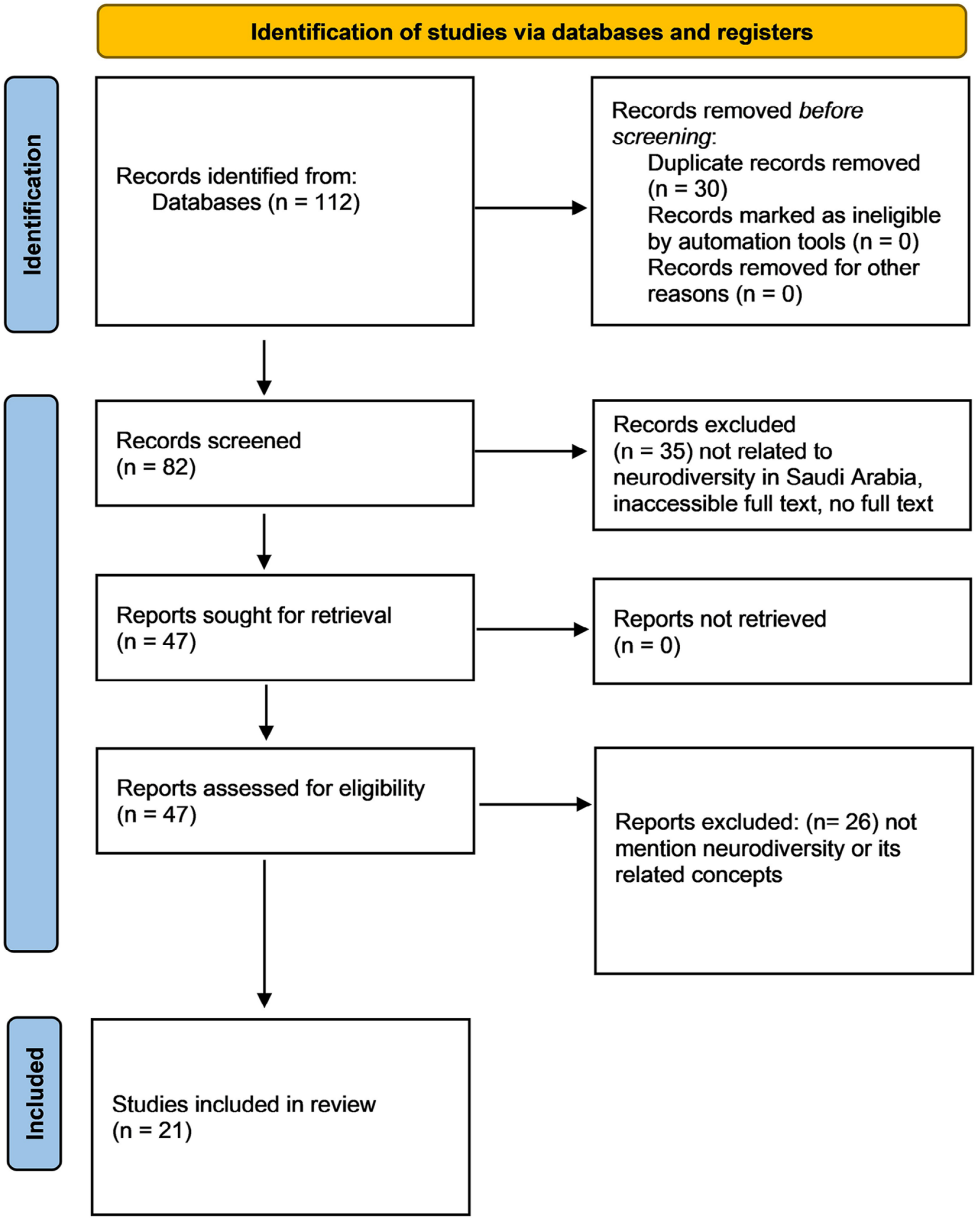
PRISMA flowchart for the inclusion and exclusion of the studies.

A screening process was conducted, focusing on titles and abstracts to assess the relevancy of each study. This process, governed by a set of inclusion criteria, reduced the pool to 21 pertinent studies. The inclusion criteria mandated that studies pertain to special education within the Saudi Arabian context and include either primary or secondary data. Studies were excluded if they referenced special education but did not focus on Saudi Arabia. The resulting sample of studies provided a robust dataset for the subsequent content analysis. See Figure 1 for the inclusion and exclusion PRISMA flowchart. Since the search terms were limited to titles, it was easier to decide on the included papers and exclude ones without any need for conflicting papers that require discussions among the authors, although an initial protocol was prepared including that any included studies should clearly discuss the inclusion of neurodiverse individuals, be it in educational and work settings. In other words, studies that only examine special education in terms of exclusion or segregation were not excluded as they promote separating the neurodiverse population from others, albeit required sometimes for severe cases.

### Instrument

The measures implemented in this content analysis were twofold. Firstly, the analysis involved the extraction of data relevant to the intersection of special education laws and neurodiversity within the selected Saudi Arabian studies. This process required a detailed examination of the content to discern the authors’ perspectives on inclusion, segregation, and neurodiversity as demonstrated in their findings and conclusions.

Subsequently, the extracted information was thematically categorized into a conceptual framework representing the current state and implications of special education laws in Saudi Arabia. This framework considered whether the laws and their implementation support or oppose the practice of neurodiversity. The studies were classified based on their explicit or implicit support, opposition, or mixed views regarding neurodiversity, with particular attention paid to their stance on inclusion and segregation.

The steps are demonstrated in table 1 and table 2, where the former included citation, aim, findings, relevance or implication to neurodiversity, and position when extracting the data, and the latter included category, elaboration, how studies accounted for the category, possible implications for neurodiversity in Saudi Arabia, and sample studies.

### Design

The research employed a content analysis study design, a methodological approach suited for the analysis of communication within the textual data. Content analysis facilitates the identification, coding, and categorizing of patterns and themes within qualitative data. It is uniquely positioned to handle large volumes of data, allowing for the distillation of information into actionable insights. This technique is particularly advantageous in policy research where the interpretation of language and legislation is critical ( Hsieh & Shannon, 2005; Vaismoradi et al., 2013; Vears & Gillam, 2022).

### Procedures

The study commenced on February 6, 2024, with an initial literature review to identify relevant studies on the topic of special education in Saudi Arabia. A team of three researchers independently reviewed the papers, ensuring an unbiased selection process. Any discrepancies in study inclusion were resolved through discussion and consensus-building among the research team members. All the data associated with this study including PRISMA flowchart, PRISMA checklist are available in our supplementary preregistered data ( Almakrob et al., 2024).

Upon finalizing the list of included papers, a structured data extraction protocol was implemented. The data were organized into two main tables designed to facilitate the synthesis of findings (see Table 1 and Table 2). These tables served as the foundation for the development of a conceptual model, which aimed to encapsulate the role of special education in promoting or impeding the practice of neurodiversity in Saudi Arabia. Three rounds of data extraction were performed. The first round included the initial extraction for each of these two tables based on the assigned themes/variables for each table. The second round required verification of the extracted data by two of the authors and providing comments. The third round included discussing the comments, which were mostly about the size of the extracted data and reducing it to make it more informative, and then the whole team agreed on the existing published data.

The synthesis of data culminated in a conceptual model that not only reflects the current legal and educational landscape but also provides insights into potential directions for policy and practice. This model serves as a tool for stakeholders in Saudi Arabia’s education sector, offering a clear depiction of the interplay between special education legislation and the principles of neurodiversity.

## Results

The results section commences with a detailed table encapsulating summaries of the selected studies, aligned with their implications for special education and the burgeoning concept of neurodiversity within the Saudi Arabian context. This initial synthesis served as the foundation for a deeper analysis, leading to the development of a subsequent table where studies were systematically categorized into seven distinct themes. These themes elucidate varying dimensions of special education laws and their intersection with neurodiversity, providing insight into whether these laws serve to enhance or hinder the practice of neurodiversity in Saudi Arabia. Drawing from the rich data of these categorizations and the understanding they afford; we have crafted a conceptual model. This model serves as a theoretical framework that captures the application of special education laws to neurodiversity in Saudi Arabia, offering a reflective lens on both the current state and potential future directions of special education practices in the Kingdom and their broader international implications. It is important to note that in this section we present our interpretation and inferences for the collected data, which are detailed in the (Extended data) link below (Table 1, Table 2, Data 1, and Data 2).

### A Synthesis for Special Education and Neurodiversity in Saudi Arabia

Table 1 presents a synthesis of 21 studies, each analysed to discern their contribution to the understanding of special education laws and their interplay with neurodiversity in Saudi Arabia ( Almakrob et al., 2024). The studies were chosen based on their explicit aim to critique, explore, and assess various aspects of special education ranging from policy analysis, educator preparedness, inclusivity in educational practices, and the readiness of schools to embrace neurodiverse students. The findings from these studies collectively underscore a growing recognition of the need for systemic shifts towards inclusive educational environments that are responsive to the diverse needs of all learners, including those with disabilities. Each study adds to the argument that special education, when effectively implemented, can be a robust vehicle for supporting neurodiversity, as it advocates for the adaptation of teaching methodologies, transition services, and educational technologies that cater to the spectrum of learning needs. The studies vary in their position, with most supporting the advancement of neurodiverse-inclusive practices, some presenting a mixed view indicating the existence of barriers to full realization, and others opposing, reflecting resistance to inclusive practices. Collectively, these academic inquiries pave the way for a conceptual model that encapsulates the potential of special education laws to support or hinder the practice of neurodiversity in Saudi Arabia, offering implications for both national educational strategies and global discourse on inclusive education.

### Categories for Special Education and Neurodiversity in Saudi Arabia

The landscape of special education in Saudi Arabia is under rigorous scholarly scrutiny, as evidenced by a multitude of studies exploring various facets of this intricate field. The purpose of this essay is to synthesize key categories derived from a content analysis of 21 studies, providing a panoramic view of special education laws and their alignment with the concept of neurodiversity in Saudi Arabia. These categories not only reveal the current state of special education but also forecast potential trajectories for the promotion of neurodiversity in the Kingdom.

One prominent category that emerges from the analysis is the Implementation of Inclusive Education, which is pivotal for fostering a neurodiverse-friendly educational environment ( Abu-Alghayth, 2021; Alsalem & Alzahrani, 2023). This category underscores the necessity to critique and improve the practical application of inclusive education, which aims to integrate students with disabilities into mainstream classrooms. It echoes the sentiment that without robust implementation strategies, the theoretical underpinnings of inclusive education policies may fail to materialize into tangible benefits for students with disabilities, potentially perpetuating segregation rather than fostering inclusivity.

Transition and Post-School Outcomes is another significant category, emphasizing the importance of equipping students with disabilities for life beyond the classroom ( Almalki, 2022; Alsalamah, 2023). This category underscores a lacuna in effective transition planning and services, suggesting that enhanced transition services could lead to greater societal acceptance and integration of neurodiverse individuals. The implications of this category are profound, as the quality of transition services directly correlates with the ability of individuals with disabilities to contribute meaningfully to society and lead fulfilling lives.

The third category, Educational Barriers and Facilitators, highlights the factors influencing the educational experiences of students with disabilities ( Abu-Alghayth, 2020; Ashur & Bagadood, 2022). The findings within this category suggest that addressing barriers such as insufficient teacher preparedness and enhancing facilitators like the use of evidence-based practices and assistive technology are essential for creating a learning environment that respects and caters to neurodiversity. This category underscores the necessity of a multifaceted approach to special education, one that considers the complex interplay of various factors affecting the educational journey of students with disabilities.

Stakeholder Attitudes and Knowledge is a category that encapsulates the spectrum of beliefs and awareness levels among individuals directly involved in or affected by special education ( Abed, 2020; Bagadood & Sulaimani, 2022). Studies in this category reveal that attitudes and knowledge of stakeholders, including educators, parents, and policymakers, can either act as formidable barriers to or facilitators of the integration of students with disabilities. This category is critical, as it underscores the importance of informed and positive stakeholder engagement for the advancement of neurodiversity.

Lastly, the category of Policy Analysis and Development is crucial as it pertains to the examination and advancement of legislation aimed at supporting students with disabilities ( Alquraini, 2014; Mohammed, 2018). This category suggests that reflective and progressive policymaking is foundational for an educational system that not only accommodates but also celebrates neurodiversity. Studies in this category provide a framework for understanding the current policy landscape and suggest pathways for legislative enhancements that can better meet the needs of students with disabilities.

### A Conceptual Model for Special Education Laws in Saudi Arabia

**Figure 2.**
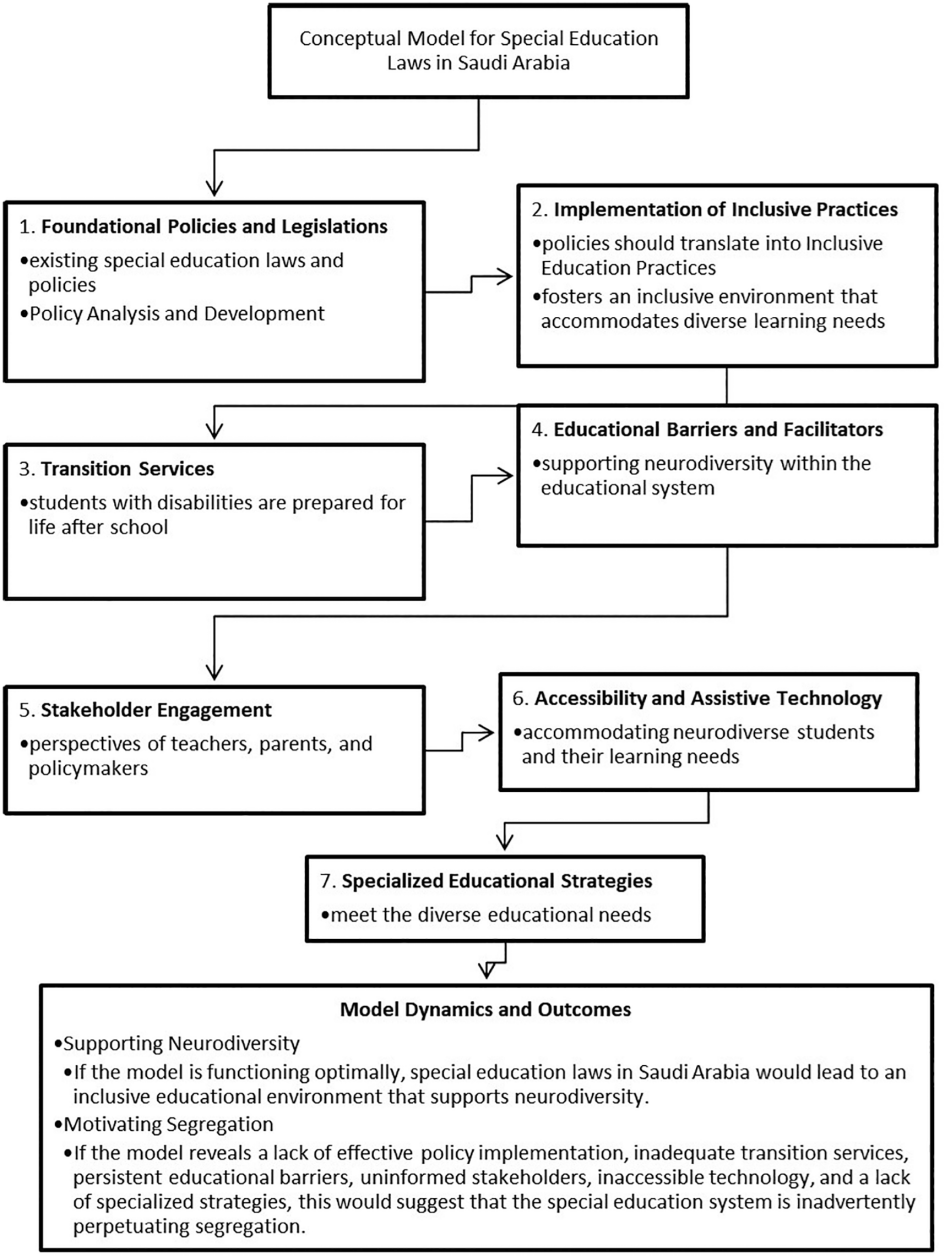
A conceptual model for special education laws in Saudi Arabia.

The conceptual model presented delineates a multifaceted approach to understanding special education laws in Saudi Arabia and their alignment with the concept of neurodiversity (see Figure 2). It provides a framework for evaluating whether the current educational landscape supports the integration of neurodiverse individuals or inadvertently contributes to their segregation. Each aspect of the model plays a crucial role in this evaluation:

1. **Foundational Policies and Legislations:** The cornerstone of the model is the robustness of special education laws and policy formulation. Policies that are inclusive, and aligned with international standards set the stage for a supportive environment for neurodiversity. However, if these policies are not well-conceived or are out of sync with global best practices, they may create systemic barriers that limit the participation of neurodiverse students ( Alquraini, 2014; Mohammed, 2018).
2. **Implementation of Inclusive Practices**: The transition from policy to practice is a critical step. Effective implementation of inclusive education practices indicates a commitment to neurodiversity, facilitating diverse learners’ access to mainstream education and promoting positive social interactions ( Abu-Alghayth, 2021; Alsalem & Alzahrani, 2023). Lack of implementation, on the other hand, can lead to de facto segregation, even if unintentional.
3. **Transition Services**: Quality transition services are indicative of a system that values neurodiverse students beyond their academic tenure, preparing them for life’s various roles. Adequate transition planning reflects an understanding that education is not an end in itself but a means to a fulfilling and autonomous life ( Almalki, 2022; Alsalamah, 2023). Without these services, students may find themselves marginalized from mainstream society and the workforce.
4. **Educational Barriers and Facilitators**: Identifying and managing barriers while leveraging facilitators is akin to navigating a ship through a narrow strait. Barriers such as inadequate teacher training or lack of resources can impede progress, while facilitators like assistive technology and evidence-based practices can accelerate the journey toward a neurodiverse-friendly educational environment ( Abu-Alghayth, 2020; Ashur & Bagadood, 2022).
5. **Stakeholder Engagement**: The attitudes and knowledge of stakeholders can dramatically shape the educational experience of neurodiverse students. Educated and positive stakeholder communities can become champions of inclusion, advocating for and reinforcing the principles of neurodiversity within the educational system ( Abed, 2020; Bagadood & Sulaimani, 2022). Conversely, uninformed or negative attitudes can create an unwelcoming environment that stifles the potential of neurodiverse individuals.
6. **Accessibility and Assistive Technology**: Accessibility is a key indicator of a system’s commitment to neurodiversity. When students with disabilities have access to appropriate assistive technologies, it can level the playing field, allowing them to fully engage with the curriculum and their peers ( Abu-Alghayth, 2020; Alsamiri et al., 2022). A lack of accessible options suggests a system that is not fully prepared to support neurodiverse students’ needs.
7. **Specialized Educational Strategies**: Tailored educational strategies recognize and cater to the individualized learning profiles of neurodiverse students. The use of specialized strategies is a testament to a system’s adaptability and its willingness to meet students “where they are” in terms of learning needs ( Alasim, 2020; Ashour & Bagadood, 2022). Without such strategies, students may not receive the support they require to thrive.

## Discussion

The aim of this study was to conduct a content analysis of the literature concerning special education in Saudi Arabia, with a particular emphasis on the concept of neurodiversity, which remains a relatively nascent idea within the Saudi context. The analysis sought to determine whether the special education laws in Saudi Arabia are conducive to fostering an environment that supports neurodiversity or whether they contribute to the segregation of neurodiverse individuals. The findings revealed that while there is a framework in place that could support neurodiversity, including policies that draw from international best practices and a growing awareness of inclusive education, there are still significant barriers to its full implementation. These barriers range from insufficient policy implementation and stakeholder attitudes to inadequate transition services and educational resources.

The results of this study align with previous literature that emphasizes the importance of teacher preparation in supporting neurodiverse students. De Arment and Traylor and Rosenblatt, Frates, and Jackson both underscore the need for special educators to be equipped with the knowledge and skills to support neurodiverse learners effectively ( De Arment & Traylor, 2023; Rosenblatt et al., 2023). This is consistent with the current study’s findings, which highlight the need for professional development and curriculum adaptation within Saudi Arabia’s special education framework. Moreover, Shenker, Rodgers, Guitar, and Onslow discuss the diverse viewpoints on neurodiversity and ableism in clinical practice, mirroring the diversity of perspectives on inclusion within the Saudi context ( Shenker et al., 2023).

Additionally, the representation of neurodivergent individuals in media, as analysed by ( Lam & Wong, 2023), plays a crucial role in shaping public discourse and understanding of neurodiversity. This has parallels with the Saudi context, where public perception and stakeholder attitudes are determining factors in the support for neurodiversity. Asbell-Clarke et al. highlight the importance of including neurodiverse learners in computational thinking activities, suggesting that educational content and methods should be adapted to support diverse cognitive abilities—a finding that resonates with the need for specialized strategies identified in this study ( Asbell-Clarke et al., 2022).

Hunt’s et al. examination of special educators’ knowledge of student mathematical thinking further supports the argument that educators must understand diverse ways of reasoning to effectively teach students with disabilities ( Hunt et al., 2021). This is in line with the current study’s findings that teacher preparedness and knowledge are crucial for implementing effective special education practices. Garner’s work on dyslexia emphasizes the need to update strengths-based practices and attitudes, paralleling the Saudi context where neurodiversity is often framed within a deficit-based perspective that needs to be shifted ( Garner, 2021).

Grinker’s (2020) analysis of autism and the sociocultural construction of stigma through an economic lens presents an interesting comparison to the Saudi context, where the conceptualization of neurodiversity could be influenced by various social and economic factors. Lambert’s critique of deficit mythologies surrounding students with learning disabilities in mathematics echoes the findings of this study ( Lambert, 2018), where there is a need to counter limiting beliefs and support neurodiverse students’ potential in Saudi Arabia. Silberman’s discussion on neurodiversity rewiring conventional thinking about brains is particularly relevant as it advocates for a focus on native strengths and special interests ( Silberman, 2017), which is a perspective that needs to be integrated into Saudi Arabia’s special education framework.

Puccini’s et al. work on educational access through multisensory design principles for neurodiverse learners ( Puccini et al., 2013) and Tincani’s et al. exploration of diversity in educational interventions for ASD ( Tincani et al., 2009) further underscore the importance of designing educational interventions that recognize and embrace neurodiversity. These studies collectively highlight that while there is a growing recognition of the need to support neurodiverse learners, there are still considerable gaps in practice and attitudes that must be bridged.

In summary, the current study’s findings are consistent with the broader literature in that they recognize the critical role of inclusive policies, educator preparation, and a shift in attitudes towards neurodiversity. However, they also reveal that Saudi Arabia, much like other contexts, faces significant challenges in fully realizing a supportive environment for neurodiverse individuals. As Saudi Arabia continues to develop its special education framework, it is imperative that the principles of neurodiversity are ingrained within all aspects of policy and practice to ensure that all learners can thrive in an inclusive and supportive educational system.

### Implications

This study holds several implications for educational policy and practice within the Kingdom of Saudi Arabia. The conceptual model developed herein provides a scaffold for policymakers to examine and refine existing special education laws, ensuring they are conducive to fostering neurodiversity. It suggests that a shift in emphasis from a deficit-based to a strengths-based perspective within special education could enhance the support for neurodiversity. For practitioners, this study underscores the importance of professional development tailored towards understanding and embracing neurodiversity in the classroom. Furthermore, it highlights the need for increased cross-cultural and cross-linguistic research to enrich the understanding of special education within the Saudi context, potentially informing a more inclusive global narrative on neurodiversity.

### Limitations

The present study, while comprehensive in its approach to content analysis, is not without limitations. The scope was confined to published literature, which inherently may not capture the entirety of the special education landscape in Saudi Arabia, including potentially influential yet unpublished policies, grey literature, or on-the-ground practices. Moreover, the analysis was limited to documents in English, potentially omitting relevant research or policy documents published in Arabic. The study also assumes that the presence of laws or policies is indicative of their implementation, which may not always be the case due to various socio-cultural, economic, or political barriers that impede actualization. Therefore, the findings should be interpreted with caution and viewed as indicative rather than exhaustive, acknowledging the potential for gaps between policy intent and practice.

## Ethical statement

Neither human nor non-human participants were included in this study, so an IRB approval was not required.

## Data Availability

The extracted data supporting this study and PRISMA checklist are accessible from OSF repository registered on 30 August 2024, available at https://doi.org/10.17605/OSF.IO/TKQG6 ( Almakrob et al., 2024). Secondary data used in this study is available at https://doi.org/10.17605/OSF.IO/TKQG6 ( Almakrob et al., 2024). Data are available under the terms of the Creative Commons 1.0 Universal License (CC0 1.0).
