# Supplementary material for: Neurodiversity in Saudi Arabia: Towards quality education and reduced inequalities
Source: F1000Res. 2024 Dec 9;13:1062. Originally published 2024 Sep 17. [Version 2] doi: 10.12688/f1000research.155002.2 (PMC11725041; doi:10.12688/f1000research.155002.2)
Supplement: Peer review history

# Reviewer report: Reviewer response for version 2

**DOI:** 10.5256/f1000research.175251.r351222

**Published:** 2025-01-11

**Authors:** Eleanor McSherry

**Affiliations:** 1 University College Cork, Cork, County Cork, Ireland

This article is clearly presented, accurately written, and well-researched, citing current and relevant literature. The design of the study is technically sound and appropriate. The methodology is described in sufficient detail, making it easy to replicate. The analysis and interpretation are thoughtfully constructed, providing a comprehensive discussion. The implications section is engaging and aligns with current international research. Additionally, the limitations addressed are reasonable and typical for this type of research.

 Overall, this is a very interesting and timely piece, particularly given the growing body of research on neurodiversity and its implications for special education and education as a whole, this discussion extends beyond the Kingdom of Saudi Arabia. This study will be a welcomed contribution to the field of education, as its insights are relevant beyond its geographical context.

 The only critique of the study is the misuse of the term “neurodiverse.” While it is commonly used, it is often misinterpreted. As noted by researchers such as Judy Singer and Nick Walker, “neurodiverse” refers to the diversity of all human brains, as all brains are neurologically diverse. The correct term to use when describing a brain that functions differently from what is considered medically “normal” is “neurodivergent.” This distinction is especially important when referring to neurodivergent individuals and the concepts of neuro-divergent friendly and neurodivergent-inclusive practices. The only time neurodiverse is used is when you are referring to a group that encompasses people who are neurotypical and neurodivergent.

Is the work clearly and accurately presented and does it cite the current literature?

Yes

If applicable, is the statistical analysis and its interpretation appropriate?

Yes

Are all the source data underlying the results available to ensure full reproducibility?

Yes

Is the study design appropriate and is the work technically sound?

Yes

Are the conclusions drawn adequately supported by the results?

Yes

Are sufficient details of methods and analysis provided to allow replication by others?

Yes

Reviewer Expertise:

neurodiversity, education, special education, autism, disability and the media

I confirm that I have read this submission and believe that I have an appropriate level of expertise to confirm that it is of an acceptable scientific standard.

# Reviewer report: Reviewer response for version 1

**DOI:** 10.5256/f1000research.170114.r337171

**Published:** 2024-11-22

**Authors:** Clayton Keller

**Affiliations:** 1 VIA Education Strategists, North Carolina, USA

The article, *Neurodiversity in Saudi Arabia: Towards quality education and reduced inequalities*, aims to consider the extent to which special education laws and policies in the Kingdom of Saudi Arabia are supportive of the concept of neurodiversity and individuals with such conditions via a content analysis of published articles about special education in the country. The study’s premise raises two questions for me. First, if neurodiversity is a relatively recent conceptualization of the conditions experienced by a range of individuals, is it fair, in a sense, to judge whether laws and policies created before either the development or at least the increased attention to the conceptualization are amenable to the conditions? Also, wouldn’t a content analysis of the country’s laws and policies themselves be a more direct assessment of neurodiversity acceptance and support than studies about special education for which they judge the studies’ authors’ perspectives about KSA’s laws and policies (if even such perspectives could be accurately determined in all cases)?

 In a number of instances, sufficient details were not provided about the study’s methodology and analysis to make the research replicable. For instance, the narrative says that 306 potential studies were found initially yet the PRISMA flowchart starts with 112. Why is there a discrepancy? No information is provided about the instrument used as part of the content analysis; its inclusion as one of the supplemental documents would have been useful (and if the instrument were the very detailed PRISMA flowchart in the supplemental materials, say so). (The authors did do a good job in providing such resources in the OSF Registry for this study.) The authors say that “the extracted information was systematically categorized into a conceptual framework” (p. 6) but no information is provided about such a systematic approach. I would urge the authors to provide many more details in any revision of the current article. Providing citations, too, for the specific content analytical approaches that were used would help other researchers conduct similar work if they were inclined.

 I would also suggest that the authors support their statements of the results with very specific details from the studies they cite in support of the results. That could strengthen the validity of their conclusions, moving beyond their present statements of generalities.

 Finally, in a revision, I would remove the many adverbs and adjectives that the authors use to present their work in a positive light, e.g., meticulously, comprehensive, thorough, nuance, and valuable, amongst others. A reader assumes that, in a published article, such qualities informed the research. To frequently state this, though, can raise doubt in the reader’s mind.

Is the work clearly and accurately presented and does it cite the current literature?

Partly

If applicable, is the statistical analysis and its interpretation appropriate?

Not applicable

Are all the source data underlying the results available to ensure full reproducibility?

Yes

Is the study design appropriate and is the work technically sound?

Partly

Are the conclusions drawn adequately supported by the results?

Partly

Are sufficient details of methods and analysis provided to allow replication by others?

No

Reviewer Expertise:

International special and inclusive education, special educator teacher preparation, special education policy

I confirm that I have read this submission and believe that I have an appropriate level of expertise to confirm that it is of an acceptable scientific standard, however I have significant reservations, as outlined above.

# Response

**Published:** 2024-11-24

**Authors:** Ahmed Alduais

**Affiliations:** College of Education, Qatar University, Doha, Doha, Qatar

*Dear Keller,*

*Thank you so much for your effort to provide this valuable peer review. We have now completed revising our paper based on your feedback. We have tried to our best to apply as much as possible of these suggestions in the new revised versions. You can see all changes in red.*

*Below you will find your comments and our responses.*

*Once again, thank you so much.*

*Ahmed Alduais (PhD)*

*Department of Psychology*

*Norwegian University of Science and Technology, Trondheim, Norway*

 The article, *Neurodiversity in Saudi Arabia: Towards quality education and reduced inequalities*, aims to consider the extent to which special education laws and policies in the Kingdom of Saudi Arabia are supportive of the concept of neurodiversity and individuals with such conditions via a content analysis of published articles about special education in the country. The study’s premise raises two questions for me. First, if neurodiversity is a relatively recent conceptualization of the conditions experienced by a range of individuals, is it fair, in a sense, to judge whether laws and policies created before either the development or at least the increased attention to the conceptualization are amenable to the conditions? Also, wouldn’t a content analysis of the country’s laws and policies themselves be a more direct assessment of neurodiversity acceptance and support than studies about special education for which they judge the studies’ authors’ perspectives about KSA’s laws and policies (if even such perspectives could be accurately determined in all cases)?

***Author Response: **Thank you, Keller. The two questions you raised are quite reasonable. Regarding the first question, it is indeed unfair to consider given the novelty of the concept. As for the second question, you are correct; however, we opted to discuss laws and policies in the introduction (literature review) and concentrate on the data from existing studies. This approach was motivated by the fact that, as you pointed out, the concept is not utilized in the Saudi context. Consequently, we applied inclusion and exclusion criteria to draw implications for the use of this concept. In light of your questions, we have acknowledged this as a limitation.*

 In a number of instances, sufficient details were not provided about the study’s methodology and analysis to make the research replicable. For instance, the narrative says that 306 potential studies were found initially yet the PRISMA flowchart starts with 112. Why is there a discrepancy? No information is provided about the instrument used as part of the content analysis; its inclusion as one of the supplemental documents would have been useful (and if the instrument were the very detailed PRISMA flowchart in the supplemental materials, say so). (The authors did do a good job in providing such resources in the OSF Registry for this study.) The authors say that “the extracted information was systematically categorized into a conceptual framework” (p. 6) but no information is provided about such a systematic approach. I would urge the authors to provide many more details in any revision of the current article. Providing citations, too, for the specific content analytical approaches that were used would help other researchers conduct similar work if they were inclined.

***Author Response: **Thank you. The number of studies has been updated. A clarification has been added to the instruments section. This is due to the journal's policy of not including tables within the manuscript, which necessitates a clearer presentation of tables and data. We have included four tables and the PRISMA checklist in the OSF, as detailed below.*

*Table 2.docx*

*Data 1 Special education laws KSA neurodiversity KSA Alduais et al..docx*

*Data 2 Studies in special education laws in the KSA neurodiversity KSA Alduais et al..docx*

*Table 1.docx*

*PRISMA_2020_checklist neurodiversity KSA Alduais et al..docx*

*We replaced the word 'systematically' with 'thematically' because our study is not actually a systematic review.*

 I would also suggest that the authors support their statements of the results with very specific details from the studies they cite in support of the results. That could strengthen the validity of their conclusions, moving beyond their present statements of generalities.

***Author Response: ** Thank you, Keller. Your emphasis on the necessity of presenting primary evidence rather than inferences is well-taken. Indeed, if you review the four tables that the journal instructed us to publish in OSF, as per their policy, you'll find all the details there. They have chosen to include only the main findings in the main manuscript. In essence, we were directed to relocate all data and tables to the extended data section in OSF. Acknowledging the value of your points, we have made a reference for readers to this perspective.*

https://osf.io/tkqg6

 Finally, in a revision, I would remove the many adverbs and adjectives that the authors use to present their work in a positive light, e.g., meticulously, comprehensive, thorough, nuance, and valuable, amongst others. A reader assumes that, in a published article, such qualities informed the research. To frequently state this, though, can raise doubt in the reader’s mind.

***Author Response: ** Thank you. We have eliminated as many adjectives and adverbs as possible.*

# Reviewer report: Reviewer response for version 1

**DOI:** 10.5256/f1000research.170114.r333967

**Published:** 2024-11-21

**Authors:** Lori Severino

**Affiliations:** 1 Drexel University, Pennsylvania, USA

**Peer Review of the Article Using Content Analysis**

 The article under review presents a comprehensive approach to a content analysis of the current state of neurodiversity and special education law and process in Saudi Arabia, effectively situating its research within the context of current scholarship. One of the strengths of this paper lies in the use of up-to-date citations and research, which underscores the authors' dedication to maintaining relevance and engaging with the most recent developments in the field. This ensures that the analysis is not only current but also integrates contemporary findings that add to the overall robustness of the study.

 A helpful feature of the article is the thoughtful development of a conceptual model. This model provides a structured framework that guides the reader through the authors' approach and findings. It holds promise as a foundational tool for further investigation by other researchers, which could lead to the expansion and refinement of existing theories and practices.

 The authors also excel in connecting their findings to the broader landscape of research. By drawing meaningful comparisons and contrasts between their results and those found in existing literature, they demonstrate a deep understanding of the field. 

 Furthermore, the flow chart included in the article is clear and concise, aiding readers in visualizing the process and results of the content analysis. This visual tool enhances comprehension and ensures that complex ideas are communicated effectively, allowing readers to grasp the study’s conclusions with ease.

 **Areas for Improvement**

 While the study design demonstrated a solid process, there are a few areas where it could be improved to enhance the clarity and replicability of the research. For instance, although the process was well-outlined, it would have been beneficial for the authors to provide more detailed information on the number of articles that required discussion among the authors during the coding process. Additionally, specifying whether a protocol was used to guide these discussions would have added to the transparency.

 Another point to consider is the inclusion of data on the number of coding iterations. Mentioning how many rounds of coding were conducted would have provided valuable insights into the thoroughness of the analysis and supported future researchers aiming to replicate the study.

 In terms of sources, while the research leveraged a solid base, the scope could have been expanded by using additional search engines and incorporating more diverse search terms. Terms such as "learning disabilities" or "autism" may have captured a wider array of relevant studies that were not identified using broader terms like "neurodiversity" or "special education."

 A review of Table 2 reveals that the studies included in the analysis were limited in their coverage of learning disabilities, with only one article addressing dyslexia—arguably the most common learning disability—at the higher education level. Including more comprehensive discussions or sources focused on learning disabilities would have strengthened the review. One article that could have been particularly fitting is *Aldousari A, et al., 2022 (Ref 1)* 

 By addressing these areas for improvement, the article's robustness and replicability could be enhanced, contributing to a more comprehensive analysis and a stronger foundation for subsequent research.

Is the work clearly and accurately presented and does it cite the current literature?

Yes

If applicable, is the statistical analysis and its interpretation appropriate?

Not applicable

Are all the source data underlying the results available to ensure full reproducibility?

Partly

Is the study design appropriate and is the work technically sound?

Partly

Are the conclusions drawn adequately supported by the results?

Partly

Are sufficient details of methods and analysis provided to allow replication by others?

No

Reviewer Expertise:

special education, learning disabilities, dyslexia, dysgraphia, explicit instruction

I confirm that I have read this submission and believe that I have an appropriate level of expertise to confirm that it is of an acceptable scientific standard, however I have significant reservations, as outlined above.

## References

[1] : Special Education for Students With Learning Disabilities in Saudi Arabia: Reality and Challenges. *https://eric.ed.gov/?id* .2022;

# Response

**Published:** 2024-11-24

**Authors:** Ahmed Alduais

**Affiliations:** College of Education, Qatar University, Doha, Doha, Qatar

*Dear Lori,*  

*Thank you immensely for the time and effort you invested in the peer review report.* *My colleagues and I greatly value your insightful comments for the enhancement of our paper. We have implemented changes based on your suggestions, which we trust align with your expectations.*  

*Once again, we extend our deepest thanks.*

*Ahmed Alduais (PhD), Department of Psychology*

*Norwegian University of Science and Technology, Trondheim, Norway*

 ---------------------

**Reviewer Comments:**

 While the study design demonstrated a solid process, there are a few areas where it could be improved to enhance the clarity and replicability of the research. For instance, although the process was well-outlined, it would have been beneficial for the authors to provide more detailed information on the number of articles that required discussion among the authors during the coding process. Additionally, specifying whether a protocol was used to guide these discussions would have added to the transparency.

***Author Response:*** *Clarifications have been added in blue to the methods section for both points.*

 ---------------------

**Reviewer Comments:**

 Another point to consider is the inclusion of data on the number of coding iterations. Mentioning how many rounds of coding were conducted would have provided valuable insights into the thoroughness of the analysis and supported future researchers aiming to replicate the study.

***Author Response:*** *A clarification has been added to the methods section in blue.*

 ---------------------

**Reviewer Comments:**

 In terms of sources, while the research leveraged a solid base, the scope could have been expanded by using additional search engines and incorporating more diverse search terms. Terms such as "learning disabilities" or "autism" may have captured a wider array of relevant studies that were not identified using broader terms like "neurodiversity" or "special education."

***Author Response:*** *A clarification has been added to the sample section, highlighted in blue.*

 ---------------------

**Reviewer Comments:**

 A review of Table 2 reveals that the studies included in the analysis were limited in their coverage of learning disabilities, with only one article addressing dyslexia—arguably the most common learning disability—at the higher education level. Including more comprehensive discussions or sources focused on learning disabilities would have strengthened the review. One article that could have been particularly fitting is *Aldousari A, et al., 2022 (Ref 1)* 

***Author Response:*** *Regarding this point, we did not include these studies by our colleagues as their papers were focused on policies and laws. However, in another paper recently published in the European Journal of Education, we incorporated two of their studies because that paper was more centered on attitudes and perspectives, which aligned with the focus of these two studies.*

*https://onlinelibrary.wiley.com/doi/10.1111/ejed.12809*
